# Children’s Education and Parents’ Cognitive Aging: Evidence From the Compulsory Schooling Law in China

**DOI:** 10.1093/geroni/igaa057.097

**Published:** 2020-12-16

**Authors:** Zhuoer Lin, Xi Chen

**Affiliations:** Yale University, New Haven, Connecticut, United States

## Abstract

This paper investigates the effect of promoting children’s education on level and trajectory of parents’ cognition. Using three waves of China Health and Retirement Longitudinal Study (2011, 2013, 2015), we decompose the longitudinal measures of parents’ cognition into baseline level of cognitive deficit and rate of cognitive decline through a standard linear mix-effect model (aged 45-100, N=9,736). Exploiting the temporal and geographic variations in the Chinese compulsory schooling law enforcement, we identify the causal effects of children’s education on parents’ baseline level of cognitive deficit and rate of cognitive decline using instrumental variable (IV/2SLS) approach. Our estimates suggest that children’s education not only reduces parents’ level of cognitive deficit (0.17 SD/year less), but also decreases their rate of cognitive decline (0.08 SD/year less). These novel evidence offers new insights into the intergenerational effects of children’s education on parents’ cognition, stresses the importance of lifting literacy in improving accuracy of cognitive diagnosing, and provides another policy option to delay cognitive aging and related diseases.

